# Prevalence of lifetime substances use among students in Ethiopia: a systematic review and meta-analysis

**DOI:** 10.1186/s13643-019-1217-z

**Published:** 2019-12-14

**Authors:** Hirbo Shore Roba, Addisu Shunu Beyene, Asnake Ararsa Irenso, Berhe Gebremichael

**Affiliations:** 1 School of Public Health, College of Health and Medical Sciences, Haramaya University, Harar, Ethiopia; 2 Research Center for Generational Health and Ageing, School of Medicine and Public Health, Faculty of Health and Medicine, University of Newcastle, Newcastle, Australia; 3 Deakin University, School of Exercise and Nutrition Sciences, Burwood, Australia

**Keywords:** Substances, Prevalence, High school, University, Meta-analysis, Ethiopia

## Abstract

**Background:**

The use of substances is a growing concern in Ethiopia, and their impacts on younger generation have been a concern of different professionals. Even though students are at high-risk of substance abuse, there is lack of comprehensive evidence for policy decision on substance use among students. Therefore, the aim of this systematic review and meta-analysis was to estimate the prevalence of common substances among students in Ethiopia.

**Method:**

A comprehensive literature searches were done from biomedical databases: PubMed/Medline, African Journal Online, HINARI, Science Direct, and Google Scholar for article published until Dcember 31, 2017, and Addis Ababa Uiversity’s electronic library search of unpublished thesis and dissertations. Two authors autonomously selected studies, extracted data, and evaluated quality of studies. The prevalence of lifetime substances use was estimated using the random effects model. *Q* and *I*^2^ statistics were computed to measure the extents of heterogeneity.

**Results:**

A total 676 study articles were identified from electronic databases, and 28 of them were included in meta-analysis. The analysis revealed that the lifetime prevalence of any substance use was 52.5% (95% CI 42.4–62.4%), khat 24.7% (95% CI 21.8–27.7%), alcohol 46.2% (95% CI 40.3–52.2%), and smoking cigarette 14.7% (95% CI 11.3–18.5%). Significant heterogeneity was observed but there was no significant publication bias. The lifetime prevalence of khat, alcohol, and cigarette smoking among high school vs university students was 22.5% (95% CI 15.2–30.7%) vs 25.1% (95% CI 21.9–28.5%), 41.4% (95% CI 22.1–62.1%) vs 47.8% (95% CI 39.9–55.7%), and 21.5% (95% CI 12.6–32.1%) vs 12.9% (95% CI 10.1–16.0%), respectively.

**Conclusion:**

This meta-analysis highlighted the extent of lifetime prevalence of any substance, khat, alcohol, and cigarettes smoking among students in Ethiopia. Significant percent of high school students have exposed to substances. Policy makers should devise and implement strictly binding regulation to curb widespread of substances around educational institution premises at national level. Priority should be given to intervention strategies that help delay first use of substance to prevent problems later in life. Besides, the problem warrants regular national-level educational institutions based studies focusing on the magnitude, trajectory, and consequences of substance use among students.

Systematic review registration: PROSPERO CRD42018082635

## Background

Psychoactive substances act on the central nervous system and change the activities of the brain. These substances have wide range of effects, including short-term changes in perceptions, mood, consciousness, and behaviors [1]. Substances such as alcohol, khat, and tobacco are widely used [2, 3], and they are leading causes of human sufferings and become important public health and socioeconomic issue globally [4–6]. Current trends showed that the use of psychoactive substances have considerably increased predominantly in developing countries [6].

In 2012, 5.9% of all global deaths and 5.1% of disability-adjusted life year were attributable to alcohol consumption [7]. Roerecke et al. reported that alcohol per capita consumption in 15 years and above in sub-Saharan Africa is higher than the global consumption rate [8]. Similarly, a quarter of the world population smoke cigarettes, and it was leading risk factor for premature death and disability in 2015. Smoking accounts for 11.5% of death and is among five leading risk factors of disability-adjusted lost life years (DALY) in 109 countries and territories in 2015 [9, 10]. Studies also showed that chronic khat consumption not only causes severe neurological, psychiatric, cardiovascular, dental, gastrointestinal, and reproductive dysfunction [11–16], but it also has adverse socioeconomic effects affecting other aspects of life [17].

Evidence showed that poor socioeconomic condition increases the risk of harmful drug use, and people living in low-income countries disproportionately affected by higher burden of substance-related disability and premature death [18]. It is also worthy to note that rapid economic, social, and cultural changes increased use of various substances in sub-Saharan Africa countries [19].

Like other sub-Saharan countries, Ethiopia is also facing a growing problem of substance use. The use of substances has long been a serious concern for various professionals in Ethiopia due to their adverse impacts on younger generation [20, 21]. Therefore, Ethiopia recognizes substance use by young people as a serious health and social problem, and students being a high-risk abusers of substance [22]. Studies revealed that khat, alcohol, and cigarette are commonly abused substances in general population [23–25], and they are also widely used substances by high school and university students in Ethiopia [26–34].

The use of substance is associated with various health risks. For instance, a study showed that the use of substance is associated with HIV infection and risky sexual behaviors [35]. Additionally, studies revealed that the use of substances poses high risk-taking behaviors among students, which results in economic, social, physical, and health complications [26–34]. Other study showed that substance use among students is associated with social phobia, poor academic performance, and the use of multiple substances in lifetime [36]. Furthermore, it has been documented that substance abuse is associated with suicidal attempt [37].

In Ethiopian, the study of the prevalence cigarette smoking dates back to 1984 [38], and that of poly-substances use, including khat among university students, was reported in 1988 [39]. Whereas prevalence of khat use among high school students was first reported in 1994 [40]. The available studies showed wide range of variations in magnitude of substances used by students in Ethiopia. For instance, the overall prevalence of lifetime use of any substances range from 28.4 to 82.7% [27, 34, 36, 41–43], whereas lifetime prevalence of alcohol was 22.4–50.2% [27]. Furthermore, several studies showed that lifetime prevalence of smoking range from 9.3 to 22.0% [26–28, 31, 44, 45]. The studies also revealed great disparities in lifetime prevalence of khat across various universities, 27.7–41.0% [27, 28, 31, 32, 40, 44–47].

Two meta-analyses done on prevalence of substance use among students have been documented recently [48, 49]. Both studies report prevalence substance among university students focusing khat, alcohol, and smoking cigarettes. However, the evidence showed that a vast majority of students initiated using substance before joining university [27, 28, 32, 36, 41, 46, 50, 51]. Additionally, the estimated of one meta-analysis [48] did not specify whether prevalence represents lifetime, recent, or current use of substance. Therefore, in order to foreward recommendation for comprehensive intervenation, it is crucial to have prevalence estimates of substance that represents both high school and university students.

A report of Ministry of Education of Ethiopia showed that 3,767,322 students were enrolled in secondary (grade 9–12) school, colleges, TVET, and universities in the 2015/2016 academic year. Of these, 2,421,163 of them were enrolled in secondary education, and 830,287 of them enrolled in higher education [52]. With ever increasing number of students being enrolled in secondary to higher education, it is essential to address the issue of substance use in order to produce productive human power that is free from substance abuse. Therefore, the aim of this review and meta-analysis was to provide comprehensive views of prevalence of different substances practiced by students enrolled in secondary schools and higher educational institutions in Ethiopia for concerned decision makers and to inform administrators to dealing with ever increasing challenges of substances.

## Methods

### Registration

This systematic review has been registered on the International Prospective Register of Systematic Reviews (PROSPERO CRD42018082635).

### Search strategy

A comprehensive literature search was done from biomedical databases: PubMed/Medline, HINARI, African Journal Online (AJOL), Science Direct, and Google Scholar. For unpublished studies, master’s thesis and PhD dissertation, the official website of Addis Ababa University’s electronic library [53] was searched. Additionally, the lists of references of eligible studies were explored to obtain additional studies. All published and unpublished article up to December 31, 2017 were included. The following search terms were used alone or in combination: substance, khat, alcohol, smoking, prevalence, students, university, and Ethiopia. For reporting, PRISMA guideline was used during systematic review [54] (Additional file 1: Table S1).

### Inclusion and exclusion

All studies done among secondary school, college, and university students in Ethiopia reporting combined lifetime prevalence of substances, or lifetime prevalence of khat, cigarette smoking, and alcohol consumption reporting in English language were included. The main outcomes of this review and meta-analysis were overall lifetime prevalence of any substance, lifetime prevalence of khat chewing, lifetime prevalence of alcohol consumption, and lifetime prevalence of smoking cigarette. Additionally, studies with cross-sectional design, having response rate ≥ 80%, used probability sampling techniques; reporting quality assurance methods and quality assessment score ≥ 50% were included. Review articles, studies employed non-probability sampling techniques, qualitative studies, studies available only as abstract with unclear outcomes, and studies conducted in non-regular (extension and summer) students were excluded.

### Quality assessment and data extraction

The Joanna Briggs Institute Meta-Analysis for Statistics Assessment and Review Instrument (JBI_MAStARI) was used for critical appraisal [55]. The manual contains appraisal checklists. Two reviewers independently assessed articles prior to inclusion in the final review using the checklists. Any disagreement which arose between the reviewers was solved by involving a third reviewer. Data were extracted independently by both authors. For each eligible article or abstract, information about author(s), the study setting, study period, sample size, sampling technique, method of data collection, response rate, age mean/range, substances (khat, alcohol, and tobacco) use measures (lifetime or ever use prevalence), and results were extracted on Microsoft excel 2010.

### Data analysis

The analysis of the evidence was based on all studies included in this review in accordance with a PRISMA guidelines. The extracted data were exported to STATA Version 13.0 statistical software package. During the meta-analysis, all selected studies were combined using random effects model [56] to estimate the pooled prevalence of substance use. The Cochran *Q* test and *I*^2^ statistics were used to test heterogeneity in pooled prevalence estimates. The subgroup data analyses were done using region of study setup, sample size, level of educations, proportion of female students, study year, year of publication, and age of participants. Meta-regression analyses were carried out to identify parameters (sample size, year of publication, female proportion, and age of participants) associated with substance use.

## Results

### Search results

**Fig. 1:**
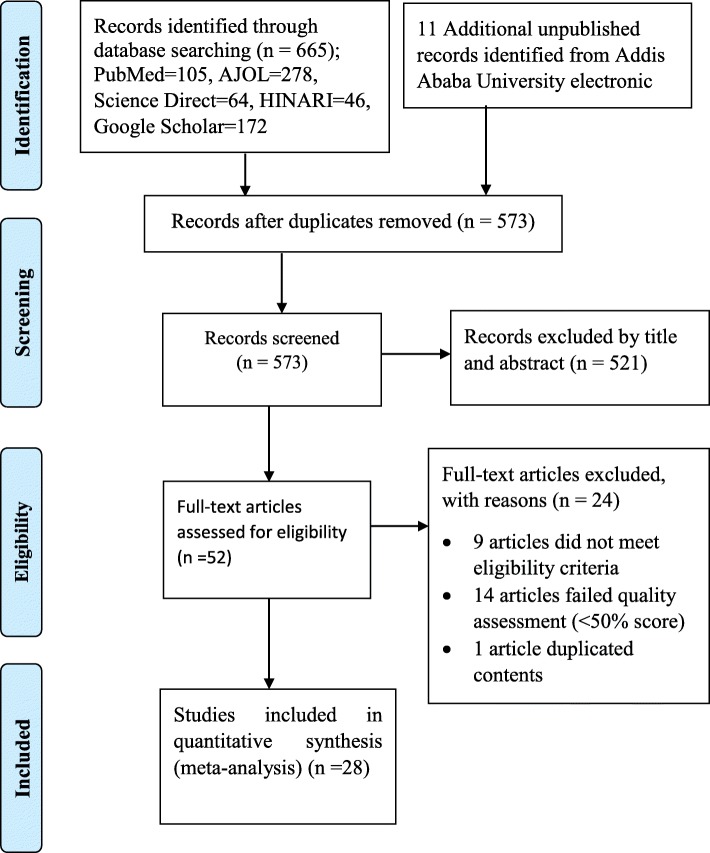
PRISMA flow chart diagram describing selection of studies for systematic review and meta-analysis on prevalence of lifetime substance use among students in Ethiopia

The review identified a total of 676 studies based on literature searches. Of these, 665 articles were from published sources and the remaining 11 were unpublished master’s thesis. From the total, 103 duplicated records were excluded and 521 records were excluded after screened by title and abstract. A total of 52 articles were screened for eligibility and quality. From these, 24 articles were excluded with reasons; 9 articles did not meet eligibility criteria, 14 articles failed quality assessment (< 50% score), and 1 article duplicated contents. Finally, 28 articles were included in the final analysis (Fig. 1).

### Characteristics of studies

The majority of the regions in Ethiopia were represented. Ten studies included in the review were from Amhara Regional State [34, 41, 43, 46, 51, 57–61], five were from Oromia regional state [27, 31, 32, 62, 63], four were from Southern Nations, Nationalities, and People’s Region (SNNPR) [33, 36, 64, 65], two were from Addis Ababa [26, 50], two were from Tigrai [28, 42], three from Harari [30, 66, 67], one from Somale Region [68], and one study done in both SNNPR and Oromia [69]. Three articles included in meta-analysis were master’s thesis [42, 60, 63]. Six studies (21.4%) were conducted between 2000 and 2010, and 20 (71.4%) studies employed stratified sampling technique. The sample size of studies included ranges from the minimum of 193, a study conducted among Mekelle University students [70] to a maximum of 3001, a study conducted among Bahir Dar University students [58]. The studies were published between 2002 and 2017 in different high schools, preparatory schools, colleges, and universities. From the studies included in the review, 9 studies conducted in high and preparatory schools [29, 30, 34, 50, 61, 62, 66, 67, 69], 21 studies conducted on university students [26–28, 31–33, 36, 41–43, 46, 47, 51, 58–60, 63–65, 68, 70], and one study conducted among polytechnic college in Debre Markos town [57] (Table 1). The proportion of females ranges from 12.5 to 56.85%. All included studies passed quality assessment based on the Joanna Briggs Institute Meta-Analysis for Statistics Assessment and Review Instrument for cross-sectional studies (JBI_MAStARI) (Additional file 2: Table S2).

**Table 1:** Characteristics of studies included in meta-analysis of prevalence of lifetime substance use among students in Ethiopia

Author year	Region/city	Study year	Study setting	Sampling technique	Mean Age (year)	Sample size	Gender *M*, *F* (%)	Response rate (%)	Khat (%)	Alcohol (%)	Tobacco (%)	Any substance (%)
Gebreslassie et al. 2013 [28]	Tigrai; Axum	2012	University	Stratified SRS	22.3	756	*M* = 60.3 *F* = 39.7	98.7	28.7	34.5	9.5	45.9
Tsegay and Esmael 2014 [55]	Amhara, Debre Markos	2013	University	SRS	21.6	800	*M* = 58.5 *F* = 41.5	94.6	30.8	35.0	11.3	48.4
Aklog T et al. 2013 [53]	Amhara; Debre Markos	2013	College	SRS	19.8	410	*M* = 54.9 *F* = 45.1	97	13.4	60.0	7.8	61.7
Kebede Y 2002 [41]	Amhara; Gonder, B/ Dar	2001	University	Stratified systematic	20.0	1103	*M* = 84.5 *F* = 15.5	87.7	26.7	-	13.1	28.4
Tesfaye G et al. 2014 [27]	Oromia; Harmaya	2013	University	Stratified SRS	20.9	1022	*M* = 76.0 *F* = 24.0	98.3	41.0	50.2	22.0	62.4
Kassa A et al. 2014 [33]	SNNP; Hawassa	2011	University	Stratified SRS	20.7	586	*M* = 81.7 *F* = 18.3	99.3	-	-	-	53.6
Kassa A et al. 2016 [61]	SNNP; Hawassa	2011	University	Stratified SRS	20.7	586	*M* = 81.7 *F* = 18.3	94.5	24.1	48.7	-	-
Fufa G et al. 2017 [66]	Somale; Jijiga	2016	University	Stratified SRS	21.2	600	*M* = 86.0 *F* = 14.0	92.6	33.3	-	-	52.4
Abrha K 2011 [42]	Tigrai; Mekelle	2011	University	Stratified SRS	20.4	601	*M =* 68.2 *F =* 31.8	90.8	35.1	69.7	17.5	82.7
Mekonen T et al. 2017 [69]	SNNP, Wolaita Sodo	2015	University	Clustered SRS	21.2	725	*M* = 66.5 *F* = 33.5	97.1	-	-	-	33.1
Adere A et al. 2017 [43]	Amhara; Woldia	2015	University	Stratified SRS	20.7	655	*M* = 69.3 *F* = 30.7	89.7	13.0	33.1	7.9	36.9
Birhanu MA et al. 2014 [34]	Amhara; Woreta	2012	GSS & PPS	Stratified SRS	17.3	651	*M* = 55.0 *F* = 45.0	95.2	34.9	59.0	22.9	65.4
Teshome G, 2012 [60]	Oromia; Adamma	2012	University	Stratified SRS	21.8	*728*	*M =* 87.5 *F =* 12.5	95.3	27.7	-	-	-
Gebrehanna E et al. 2014 [54]	Amhara; Bahr Dar	2012	University	Stratified SRS	21.2	3001	*M* = 77.6 *F* = 22.4	84.4	24	-	-	-
Wondimu GA et al. 2017 [46]	Amhara; Gondar	2011	University	Stratified SRS	21.0	736	*M* = 76.5 *F* = 23.5	92.0	27.7	-	-	-
Abdeta T et al. 2017 [32]	Oromia; Jimma	2016	University	Stratified SRS	21.9	619	*M* = 75.0 *F* = 25.0	95.1	26.3	-	-	-
Astatkie A et al. 2015 [62]	SNNP; Hawassa	2014	University	Stratified SRS	21.4	1255	*M* = 73.9 *F* = 26.1	97.3	22.8	59.9	14.8	-
Deressa & Azazh, 2011 [26]	Addis Ababa, AAU	2009	University	All	20.4	622	*M* = 68.5 *F* = 31.5	98.4	14.1	31.0	8.7	-
Dachew BA et al. 2015 [56]	Amhara; Gondar	2014	University	Stratified SRS	21.3	836	*M* = 64.4 *F* = 35.6	95.8	17.9		-	-
Deresse A et al. 2014 [31]	Oromia, Haramaya	2011	University	Stratified SS	21.0	725	*M* = 60.4 *F* = 39.6	95.0	30.3	41.7	-	-
Birhanu B, 2014 [57]	Amhara, D/Berhan	2014	University	Stratified SRS	21.2	346	*F* = 60.1, *F* = 39.9	95.0	25.4	62.7	19.6	-
Dires E, et al. 2016 [59]	Oromia; Jimma	2015	GSS	Stratified SRS	16.05	296	*M* = 43.2 *F* = 56.8	100	15.9	-	-	-
Lakew A, et al. 2014 [58]	Amhara, Ataye	2014	GSS and PPS	Stratified SRS	17.21	332	*M* = 53.6 *F* = 46.4	88.0	15.4	-	-	-
Reda A et al. 2012 [64]	Harari, Harar	2010	GSS and PPS	Cluster sampling	16.4	1721	*M* = 50.1 *F* = 49.9	91.1	24.2	-	-	-
Reda A et al. 2012 [30]	Harari, Harar	2010	GSS and PPS	Cluster sampling	16.4	1721	*M* = 50.1 *F* = 49.9	91.1	-	22.2	-	-
Reda A et al. 2012 [65]	Harari, Harar	2010	GSS and PPS	Cluster sampling	16.4	1721	*M* = 50.1 *F* = 49.9	91.1	-	-	12.4	-
Teshome and Gedif. 2013 [63]	Addis Ababa	2010	GSS and PPS	Cluster sampling	16.93	2551	*M* = 45.2 *F* = 54.8	92.4	-	45.7	-	-
Dereje N et al. 2014 [67]	Oromia and SNNP	2014	GSS and PPS	Stratified SRS	15.6	1673	*M* = 47.7 *F* = 52.3	98.2	-	-	28.6	-

*AAU* Addis Ababa University, *SNNP* Southern Nations, Nationalities and People’s Region, *GSS* General Secondary School, *PPS* Preparatory School, *B/Dar* Bahir Dar, *D/Berhan* Debre Berhan, *SS* systematic sampling, *SRS* sampling random sampling, *M* male, *F* female

### Lifetime prevalence of any substance

**Fig. 2:**
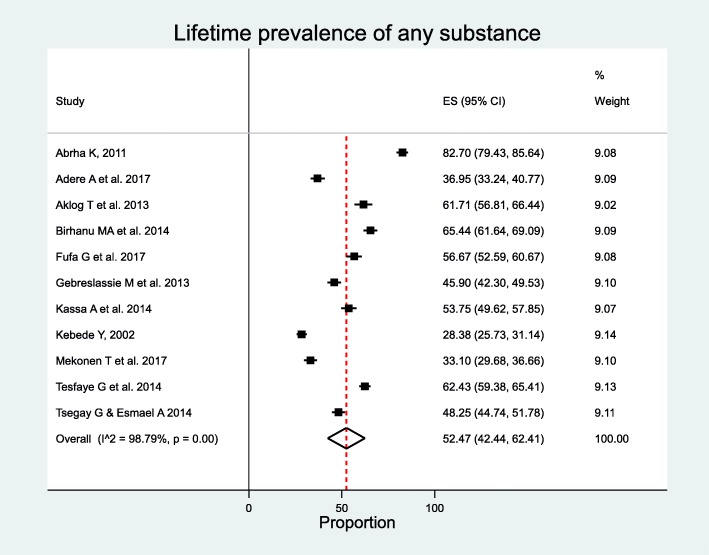
Forest plot of lifetime prevalence of any substance use among students in Ethiopia

A total of 11 studies reported lifetime prevalence of any substance use (khat, alcohol, or cigarette smoking) [27, 28, 33, 34, 36, 41–43, 51, 57, 68] with a total of 7,909 participants included in meta-analysis. The prevalence ranging from 28.4% in study conducted among Bahir Dar and Gonder University students [41] to 82.7% in study conducted in Mekelle University students [42]. The overall pooled lifetime prevalence of any substance use the use of at least one substance was 52.5% (95% CI 42.4, 62.4%). The analysis revealed substantial heterogeneity across studies with *I*^2^ = 98.8%, *p* < 0.00 (Fig. 2). However, both Begg’s test *p* < 0.1195 and Egger’s test *p* < 0.1075 showed non-significant publication bias.

Subgroup analyses were done to explore sources of heterogeneity by aggregating studies by region, sample size, female proportion, publication year, and participants’ mean age. Subgroup analysis showed that the highest prevalence was observed in Tigrai (*n* = 2), 63.3% (95% CI 60.7–65.9%), and the lowest was observed in Southern Nations, Nationalities, and People’s Region (*n* = 2), 42.2% (95% CI 39.5–44.9%). Subgroup analysis by sample size revealed that the prevalence was higher in studies with participants ≤ 1000 (*n* = 9), 54.1% (95% CI 43.7–64.4%). The prevalence was highest in studies with female proportion 40–50% (*n* = 3), 57.7% (95% CI 44.9–70.0%) and studies conducted and published between 2011 and 2014 (*n* = 7), 60.4% (95% CI 50.7–69.7%). The prevalence was higher (*n* = 2), 64.0% (95% CI 61.1–66.9%) among studies with participants’ average age younger than 20 years. Inter-group heterogeneity was observed in regions, participants’ average age, and year of publication (Table 2). However, in meta-regression analysis, year of publication, study year, sample size, proportions of female, and mean age were not associated with lifetime use of at least one substance (Table 3).

**Table 2:** Subgroup analysis of lifetime prevalence of any substance use among students in Ethiopia

Subgroup	Number of studies (*n*)	Prevalence (95% CI)	Between group heterogeneity statistics		
			Cochrane *Q*	*p* value	*I*^2^ (%)
Region					
Tigrai	2	63.3 (60.7, 65.9)			
Oromia	1	62.4 (59.4, 65.4)			
Somale	1	56.7 (52.6, 60.7)	149.5	0.000	99.0
Amhara	5	48.0 (33.7, 62.4)			
SNNP	2	42.2 (39.5, 44.9)			
Sample size					
< 1000	9	54.1 (43.7, 64.4)	3.2	0.075	99.0
1000+	2	44.4 (42.3, 46.6)			
Female proportion					
10–20	3	46.0 (27.2, 65.4)			
20–30	3	49.2 (34.8, 63.8)	2.1	0.556	98.8
30–40	2	56.7 (54.0, 59.4)			
40–50	3	57.7 (44.9, 70.0)			
Mean age					
< 20	2	64.0 (61.1, 66.9)	5.6	0.018	98.8
20+	9	50 (38.7, 61.3)			
Publication year					
2000–2010	1	28.4 (25.7, 31.1)			
2011–2014	7	60.4 (50.7, 69.7)	42.5	0.000	98.8
2015–2017	3	42.1 (28.5, 56.3)			
Study year					
2000–2010	1	28.4 (25.7, 31.1)			
2011–2014	7	60.4 (50.7, 69.7)	2.2	0.137	98.8
2015–2017	3	42.1 (28.5, 56.3)

**Table 3:** Meta-regression analysis of study level covariates of lifetime prevalence of any substance, khat, alcohol, and cigarettes smoking among students in Ethiopia

Substance/study characteristics	Coefficient (*β*)	Intercept	Standard error	*p* value
Lifetime use of any substance				
Year of publication	0.0060609	− 11.68013	0.0124317	0.638
Study year	13.89607	− 0.0082915	9.605409	0.182
Sample size	− 0.0003494	0.7741379	0.0002403	0.180
Female proportion	0.0037158	0.4131199	0.0045819	0.438
Mean age	− 0.0387067	1.317451	0.0389781	0.347
Lifetime khat use				
Study year	− 0.0020241	4.325688	0.0052509	0.704
Year of publication	− 0.0021067	4.495044	0.0051577	0.687
Sample size	0.0000108	0.2432449	0.0000275	0.698
Female proportion	− 0.100255	0.2853794	0.11553259	0.526
Mean age	0.0084523	0.0811581	0.0094737	0.383
Lifetime alcohol use				
Year of publication	0.0042828	− 8.160433	0.0242541	0.863
Study year	0.0255544	− 50.95082	0.022354	0.275
Sample size	0.0000688	0.5277942	0.0000622	0.319
Female proportion	− 0.0035171	0.5924492	0.0042616	0.425
Mean age	− 0.005842	0.3477194	0.0223419	0.798
Lifetime tobacco smoking				
Year of publication	0.0018767	− 3.622797	0.0055397	0.741
Study year	0.0035468	− 6.980033	0.0053445	0.521
Sample size	0.0000605	0.097251	0.0430878	0.171
Female proportion	0.0014625	0.1013142	0.0017218	0.414
Mean age	− 0.0173243*	0.4966166	0.0075743	0.043

*Significant association; *p* < 0.043

### Prevalence of khat use

**Fig. 3:**
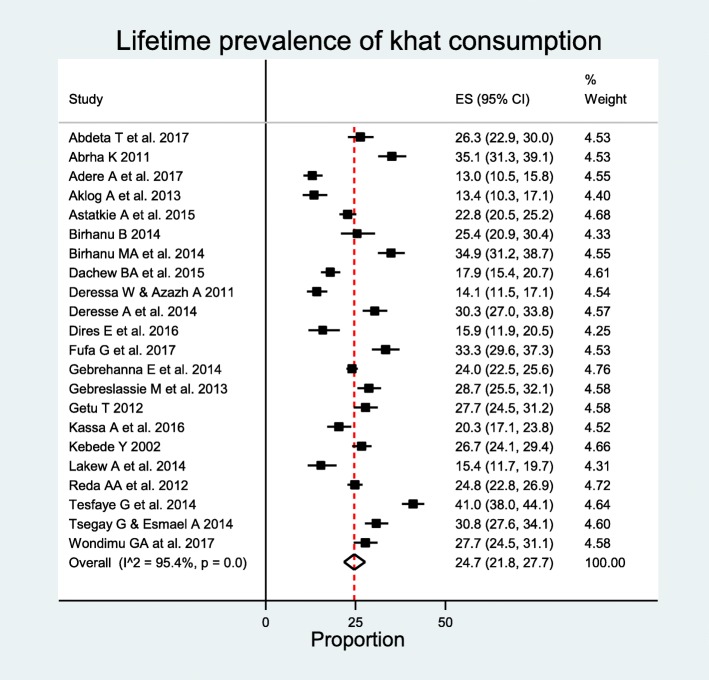
Forest plot of prevalence of lifetime khat consumption among students in Ethiopia

Twenty two articles: 19 published articles [26–28, 31, 32, 34, 41, 43, 46, 51, 57–59, 64–66, 68] and three masters theses [42, 60, 63], with a total of 17,773 participants, of this 4,621 lifetime khat users, were included in the analysis. The prevalence of lifetime khat widely varied across studies. The lowest lifetime prevalence was 13% reported in study conducted in Woldia University students [43], while the highest lifetime prevalence was 41% reported in study conducted in Haramaya University students, Eastern Ethiopia [27]. Meta-analysis of all 22 studies yielded the overall pooled prevalence of lifetime khat use, 24.7% (95% CI 21.8–27.7%). Substantial heterogeneity was observed between studies; *I*^2^ = 95.4%, *p* < 0.00 (Fig. 3**)**. However, the analysis showed that there was no significant publication bias with Egger’s test *p* < 0.1057.

Subgroup analyses were done by region, sample size, proportion of female students, level of education, and year of publication to explore for sources of heterogeneity. Accordingly, the highest lifetime prevalence of khat use was observed in study from Somale region (*n* = 1), 33.3% (95% CI 29.6–37.3%), followed by Tigray region (*n* = 2), 31.5% (95% CI 29.0–34.0%), and the lowest prevalence was observed in Addis Ababa (*n* = 1), 14.1% (95% CI 11.5–17.1%). The highest prevalence was also observed in studies with more than 1000 study participants, 27.6% (95% CI 22.3–33.2%), and it was lowest in studies with study participants less than 500, 17.3% (95% CI 12.5–22.7%). Similarly, the prevalence was highest in studies with female proportion 20–30%, 28.2% (95% CI 22.2–34.6%), and the lowest, 23.1% (95% CI 17.9–28.8%) was observed in studies with female proportion 40–50%. Subgroup analysis by level of education showed that the prevalence was higher, 25.1% (95% CI 21.9–28.5%) in studies conducted in university or college students. Subgroup analysis by age showed that prevalence was higher in studies with average age of participants 20 years or older (*n* = 17), 25.9% (95% CI 22.6–29.3%). Prevalence was also highest (*n* = 1), 26.7% (95% CI 24.1–29.4%) in a study published before 2010. The highest prevalence (*n* = 14), 26.3% (95% CI 22.6–30.3%) was also observed in studies conducted between 2011 and 2014 (Table 4). There was significance between group heterogeneity in regions and sample size (Additional file 3: Table S3). Meta-regression analysis showed that study year, year of publication, sample size, female proportion, and mean age were not significantly associated with lifetime prevalence of khat use (Table 3).

**Table 4:** Subgroup analysis of lifetime prevalence of alcohol, khat, and cigarettes smoking among students in Ethiopia

Subgroup	Khat		Alcohol		Cigarette smoking	
	*n*	% (95% CI)	*n*	% (95% CI)	*n*	% (95% CI)
By region						
Somale	1	33.3 (29.6, 37.3)		-	-	-
Tigrai	2	31.5 (29.0, 34.0)	2	50.2 (47.5, 52.9)	2	12.8 (11.1, 14.6)
Oromia	5	28.1 (21.0, 35.8)	2	46.6 (44.3, 49.0)	1	22.0 (19.5, 24.7)
Harari	1	24.8 (22.8, 26.9)	1	21.6 (19.7, 23.6)	1	14.0 (12.4, 15.7)
Amhara	10	22.6 (18.6, 26.8)	5	49.8 (36.8, 62.8)	6	13.3 (9.1, 18.1)
SNNP	2	22.0 (20.1, 23.9)	2	56.4 (54.1, 58.6)	1	14.8 (12.9, 16.9)
Addis Ababa	1	14.1 (11.5, 17.1)	2	42.5 (40.8, 44.2)	1	8.7 (6.6, 11.2)
Other	-	-	-	-	1	17.6 (11.2, 24.1)
Sample size						
< 500	4	17.3 (12.5, 22.7)	2	61.2 (57.7, 64.7)	2	12.7 (10.4, 15.1)
500–1000	13	25.8 (21.7, 30.1)	8	44.0 (34.7, 53.6)	6	12.5 (8.5, 17.2)
> 1000	5	27.6 (22.3, 33.2)	4	43.8 (28.0, 60.3)	5	18.2 (12.6, 24.5)
Female proportion (%)						
10–20	3	24.9 (20.8, 29.3)	1	48.6 (44.5, 52.8)	1	13.1 (11.2, 15.3)
20–30	5	28.2 (22.2, 34.6)	2	55.6 (53.5, 57.6)	2	17.9 (16.4, 19.5)
30–40	7	23.6 (16.9, 31.0)	5	47.6 (32.6, 62.9)	4	12.9 (7.8, 19.2)
40–50	7	23.1 (17.9, 28.8)	6	42.2 (30.5, 54.3)	5	12.7 (8.8, 17.3)
> 50	-	-	-	-	1	28.6 (26.5, 30.9)
Education level						
Secondary school	4	22.5 (15.2, 30.7)	3	41.4 (22.1, 62.1)	3	21.5 (12.6, 32.1)
University/college	18	25.1 (21.9, 28.5)	11	47.8 (39.9, 55.7)	10	12.9 (10.1, 16.0)
Mean age (year)						
< 20	5	20.5 (13.8, 28.2)	4	46.0 (28.6, 63.9)	4	17.7 (9.8, 27.3)
20+	17	25.9 (22.6, 29.3)	10	46.6 (38.3, 55.0)	9	14.7 (11.3, 18.5)
Year of publication						
2000–2010	1	26.7 (24.1, 29.4)	-	-	1	13.1 (11.2, 15.3)
2011–2014	13	26.2 (22.3, 30.4)	10	44.9 (35.7, 54.3)	10	15.7 (11.4, 20.5)
2015–2017	8	21.9 (17.6, 26.5)	4	50.1 (37.9, 62.4)	2	12.3 (10.8, 13.8)
Study year						
2000–2010	3	21.7 (15.4, 28.7)	3	32.4 (17.5, 49.3)	3	12.0 (9.2, 15.0)
2011–2014	14	26.3 (22.6, 30.3)	10	52.1 (44.6, 59.6)	9	16.6 (12.1, 21.7)
2015–2017	5	21.9 (15.5, 29.0)	1	33.1 (29.5, 36.9)	1	7.9 (6.0, 10.3)

### Prevalence of alcohol consumption

**Fig. 4:**
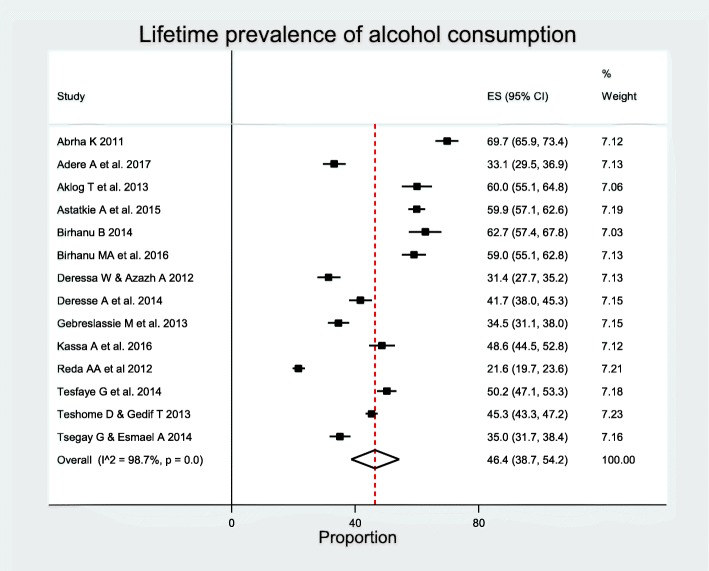
Forest plot of lifetime prevalence of alcohol consumption among students in Ethiopia

A total of 14 studies: 12 published [26–28, 30, 31, 34, 43, 50, 51, 57, 64, 65] and two master’s theses [42, 60] with the total of 12,701 participants, of which 5,598 lifetime alcohol users were included in the analysis. Lifetime prevalence of alcohol use range from 22.0% reported in study conducted among high school students in Harar [30] to 70.0% reported among Mekelle University students [42]. The studies were conducted in 2011 to 2017. The overall pooled prevalence of lifetime alcohol use was 46.4% (95% CI 38.7–54.2%). There was substantial heterogeneity with *I*^2^ *=* 98.7% and *p* < 0.00 (Fig. 4), although there was no significant publication bias with Begg’s test, *p* < 0.6614, and Egger’s test, *p* < 0.5485.

Subgroup analysis showed that the highest prevalence (*n* = 2), 56.4% (95% CI 54.2–58.7%) was observed in SNNPR and the lowest was observed in Harar (*n* = 1), 21.6% (95% CI 19.7–23.6%). The prevalence was highest in studies with sample size less than 500 (*n* = 2), 61.2% (95% CI 57.7–64.7%), female proportion 20–30% (*n* = 2), 55.6% (95% CI 53.5–57.6%) and lowest (*n* = 6), 42.2% (95% CI 30.5–54.3%) in studies with female proportion 40–50%. The prevalence was higher in studies conducted in university/college students (*n* = 11), 47.8% (95% CI 39.9–55.7%). The prevalence was also higher in studies with average age of participants 20 years or older (*n* = 10), 46.6% (95% CI 38.3–55.0%). Subgroup analysis showed that the prevalence was higher (*n* = 4), 50.1% (95% CI 37.9–62.4%) in studies published from 2015 to 2017. Highest prevalence was observed in the studies conducted between 2011 and 2014 (*n* = 10), 52.1% (95% CI 44.6–59.6%) (Table 4). There was significant heterogeneity between regions, sample size groups, and study years (Additional file 3: Table S3). Meta-regression analysis revealed that publication year, sample size, female proportion, and mean age were not significantly associated with lifetime alcohol use (Table 4).

### Lifetime prevalence of cigarettes smoking

**Fig. 5:**
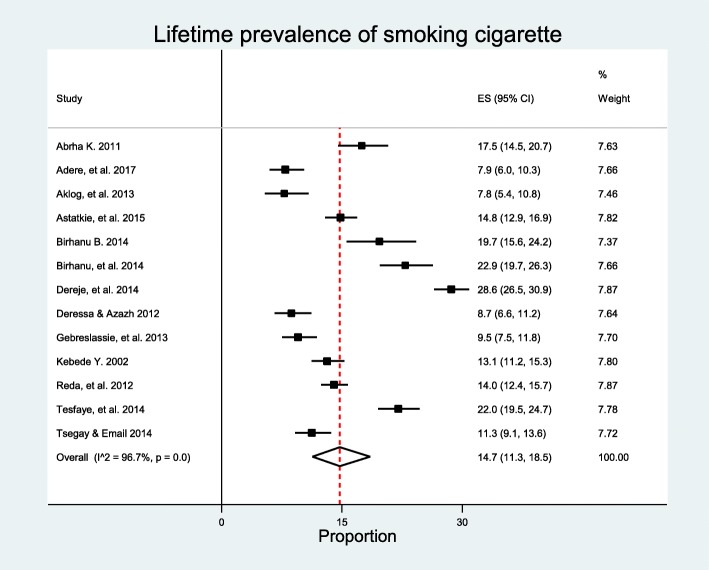
Forest plot of lifetime prevalence of cigarettes smoking among students in Ethiopia

A total of 13 studies: eleven published studies [26–28, 34, 41, 43, 51, 57, 65, 67, 69] and two unpublished studies [42, 60] with a total of 11,615 participants, of which 1,898 lifetime smokers were included in the analysis. The highest prevalence, 28.6%, was reported in adolescents in high and preparatory school in Hawassa and Jimma [69], whereas the lowest prevalence, 7.8%, was reported in study conducted among Debre Markos Polytechnic College students [57]. The pooled lifetime prevalence of smoking cigarettes among students in Ethiopia was 14.7% (95% CI 11.3–18.5). The analysis showed considerable heterogeneity among studies with *I*^2^ = 96.7% and *p* < 0.000 (Fig. 5). However, the analysis revealed that there was no significant publication bias with Egger’s test *p* < 0.269.

Subgroup analysis showed that highest prevalence was observed in studies conducted in Oromia Region (*n* = 1), 22.0% (95% CI 15.5–24.7%). But, lifetime prevalence based on six studies was from Amhara Region (*n* = 6) 13.3% (95% CI 9.1–18.1%). Subgroup analysis showed that prevalence was highest (*n* = 5), 18.2% (95% CI 12.6–24.5%) in studies with sample size larger than 1000. The highest prevalence was (*n* = 1), 28.6% (95% CI 26.5–30.9%) observed in studies with female proportion 50% or higher followed by the 17.9% (95% CI 16.4–19.5%), the estimate based on studies (*n* = 2) with female proportion 20–30%, and the lowest was (*n* = 5), 12.9% (95% CI 8.8–17.3%) in studies with female proportion 40–50%. Analysis showed that the prevalence was higher in studies conducted among secondary schools (*n* = 3), 21.5% (95% CI 12.6–32.1%) and studies with average age of study participants younger than 20 years (*n* = 4), 17.7% (95% CI 9.8–27.3%). Prevalence was highest (*n* = 10), 15.7% (95% CI 11.4–20.5%) in studies published between 2011 and 2014. When studies grouped by study years, the prevalence was highest (*n* = 9), 16.6% (95% CI 12.1–21.7%) (Table 4). Heterogeneity between groups was observed in regional, female proportion, and study year subgroup analysis, *p* < 0.00 (Additional file 3: Table S3). However, meta-regression analyses revealed that only average age was significantly associated with lifetime prevalence of tobacco smoking, *p* < 0.043 with slope, − 1.017, and intercept = 0.497 (Table 3).

## Discussion

This meta-analysis tried to estimate the pooled lifetime prevalence of most commonly used substances, khat, alcohol, and smoking cigarettes, and the overall prevalence of any substance in students in Ethiopia.

In this meta-analysis, the pooled estimates showed that more than one in two (52%) students involved in the use of at least one substance in their lifetime. The finding was consistent with the results of meta-analysis done on the lifetime prevalence of alcohol consumption among young people in eastern Africa which was 50% [71]. This indicates the use of substance is far more common among students in Ethiopian. It is not surprising to observe high prevalence of substance use when educational institutions surrounded by substance sellers, who even provide their customers with private rooms [72]. Additionally, this could be due to the facts that these substances (khat, alcohol, and smoking) are not controlled, and educational institutions do not have binding law that prevents the use of these substances in Ethiopia. Furthermore, studies [73, 74] showed ever increasing partying which is becoming the integral part of culture among students, might explain high lifetime prevalence of substances.

There was significant regional variation with the highest prevalence observed in Tigrai region, although all regions were not represented, including Oromia, the largest region. The differences could be explained by difference in social values attached to different substances [20]. Alcohol drink in Northern and khat use in southwest and Eastern part of Ethiopia are widely accepted. Additionally, the observed difference may be explained by the effect of school environment on healthy behavior, which influenced further by compositional and contextual factors [75, 76]. Similarly, there was significant variation of prevalence when studies grouped by year of publication with the highest prevalence observed in conducted 2010–2014, 60%. The use of substances affects wider range of aspects of life. The literature showed that students involved in substance use are at higher risk of developing violent behaviors, risky sexual behaviors, and withdrawal symptoms [20, 32, 77].

The pooled prevalence showed that 24.7% (95% CI 21.8–27.7%) of students had used khat at least once in their lifetime. This finding was slightly higher than national prevalence of lifetime khat consumption, 19% [78]. The observed difference could be due to difference in study population and study settings. This study showed that khat consumption is spreading at an alarming rate in recent days among students that can be attributed to misconception that khat consumption improves academic performance which in fact disagree with available literatures [36, 79]. Even, the results of meta-analysis of various studies revealed that acute or sub-chronic exposure to khat impair short-term memory [80]. The ever increasing khat consumption complemented by absence of laws that regulate the production, distribution, and use in school environment in Ethiopia, and inevitably, there will be challenges ahead in formulating rules and regulation since khat becomes one of the leading commodities for export in recent days [39], and farmers are increasingly abandoning other crops for cultivating khat [81].

Segregation of the study by region and sample size revealed substantial heterogeneity. The pooled estimate based on two studies showed that lifetime prevalence of khat use was highest in Somale Region (*n* = 1), 33.3% (95% CI 29.6, 37.3) and followed by Tigrai Region which is non-khat growing region in the country. The widespread use be explained by the fact that the region is predominantly inhabited by Muslim and khat consumption is widely accepted [82]. For the case of the Tigrai Region, the higher prevalence would be explained by the fact that khat consumption is spreading to most major cities from traditional khat growing regions [81]. The prevalence was also highest in studies with sample size greater than 1000 (*n* = 5), 27.76% (95% CI 22.59, 32.92). This indicates that the estimate was influenced by larger sample size studies.

The pooled estimate of lifetime prevalence of alcohol consumption in this meta-analysis was 46.4% (95% CI 38.7–54.2). This finding was slightly lower than national prevalence, 49% [78], and 82% in university students in eastern African countries [71]. The observed difference could be explained by difference in study population and settings. Additionally, the difference could also be attributed to social influence, peer pressure [83], and cultural differences. Most importantly, aggressive commercial promotions of various alcohol brands targeting younger people through major private and government owned media outlets are partly responsible for high consumption of alcohol among students [84, 85]. Subgroup analysis by region showed that prevalence of alcohol consumption was significantly different across regions. The variation could be attributed to difference in the social value of alcohol, settings, and study methods [20]. The prevalence significantly varied with sample size that the highest was observed in small sample size studies. However, the overall estimate was not significantly affected by sample size. Similarly, the highest prevalence was observed in studies female proportion 20–30% of sample size. This could be due to difference in risk of substance use between male and female. This could be due to the fact that being male is associated with higher risk of substances use [26, 27, 29, 30, 51, 57, 64, 86].

The pooled lifetime prevalence of cigarette smoking in this meta-analysis was 14.7%. The finding was higher than the results of EDHS analysis; 4.1% all forms of tobacco use and 8.1% prevalence in men [87]. However, the prevalence was lower than the result of meta-analysis of studies conducted in Iranian university male students, which was 19.8% [88]. The difference could be explained variation in definition of cigarettes smoking. The latter study did not specify the prevalence as current or lifetime. Additionally, the variation could be attributed to difference in population. This meta-analysis included studies conducted in secondary schools and universities whereas the EDHS conducted among adults, and meta-analysis result from Iran was based on studies done among male university students. Furthermore, cultural and socioeconomic differences might have played role. The observed between group difference in regions in Ethiopia in this meta-analysis was similar with study conducted among adults [87]. Meta-regression analysis in current study showed that age was associated with tobacco smoking, which was consistent with prevalence studies done in Ethiopia [87, 89]. However, the review by Wicki, et al. [90] argued that the results on relation of alcohol consumption and the age of the students were inconsistent.

Overall, the results of this meta-analysis showed significant number of students exposed to various substances before joining university. Early exposure to substances at younger age has adverse health and behavioral effects during adulthood [91]. For instance, studies showed that early exposure is associated with risky behaviors and sexual-transmitted diseases, early pregnancy, low educational attainment, alcohol abuse and dependency, and anti-social behavior [92–94]. Furthermore, a recent longitudinal study in Finland among substance use discordant twin demonstrated that early exposure to substance disrupts transition into adulthood [95].

The strength of this meta-analysis was the representativeness of the estimates since we strictly followed the PRISMA guideline. The other strength was various substances that are commonly used by students in Ethiopian were comprehensively presented to facilitate accessibility of the evidences for concerned decision makers. Moreover, data extractions were carried out by using comprehensive tools, and two authors independently extracted data to reduce potential risks.

However, some subgroup estimates for prevalence of any substance, khat, alcohol, and tobacco smoking based on a single study do not necessarily reflect the actual context. This limits the generalizability of the finding based on a single study estimate. Even though universities and secondary schools were represented in this study, there were limited studies representing third generation or newly established universities. Inadequate or absence of studies representing public and private colleges also limits the generalizability of the finding to all education settings. Additionally, the current meta-analysis focused on lifetime prevalence of substance use and the results do not show the current substance use status, though large majority of ever substance users are current users. Lastly, we did not pool the estimate for the risk factors because of differences in risk factors across studies. Therefore, future studies should focus on substance use risk factors.

## Conclusion

The pooled estimates of this meta-analysis highlighted the extent of lifetime prevalence of any substance, khat, alcohol, and cigarette smoking among students in Ethiopia. The uses of these substances are common in educational institutions and vary with study characteristics such as region, proportion of female students, mean age, and publication year. Therefore, policy makers should devise and implement strictly binding regulation to curb widespread use of substances around educational institution premises at national level. Priority should be given to intervention strategies that help delay first use of substance to prevent problems later in life. Besides, the issue warrants regular national-level educational institutions based studies focusing on the magnitude, trajectory, and consequences of substance use among students.

## Supplementary information

**Additional file 1:** **Table S1.** Prisma 2009 Checklist.

**Additional file 2: Table S2.** Mythological quality assessment results of studies included in the meta-analysis using the Joanna Briggs Institute Meta-Analysis for Statistics Assessment and Review Instrument (JBI_MAStARI).

**Additional file 3: Table S3.** Subgroup analysis of lifetime prevalence alcohol, khat and cigarette smoking among students in Ethiopia.

## Abbreviations

AAU: Addis Ababa University
AJOL: African Journal Online
CI: Confidence interval
DALY: Disability-adjusted life year
EDHS: Ethiopian Demographic and Health Survey
SNNPR: Southern Nations Nationalities and People’s Region
HIV: Human immuno-deficiency virus
TVET: Technical and vocational education training
