# Supplementary material for: Prevalence of lifetime substances use among students in Ethiopia: a systematic review and meta-analysis
Source: Syst Rev. 2019 Dec 14;8:326. doi: 10.1186/s13643-019-1217-z (PMC6911280; doi:10.1186/s13643-019-1217-z)
Supplement: Supplementary file 2 — Additional file 2: Table S2. Mythological quality assessment results of studies included in the meta-analysis using the Joanna Briggs Institute Meta-Analysis for Statistics Assessment and Review Instrument (JBI_MAStARI). [file 13643_2019_1217_MOESM2_ESM.docx]

**Quality assessment for results**

Table S2: Mythological quality assessment using The Joanna Briggs Institute Meta-Analysis for Statistics Assessment and Review Instrument (JBI_MAStARI)

| **S.No** | **author** | **Q1** | **Q2** | **Q3** | **Q4** | **Q5** | **Q6** | **Q7** | **Q8** | **Q9** | **Q10** | **Score (%)** | **Decsion** |
| --- | --- | --- | --- | --- | --- | --- | --- | --- | --- | --- | --- | --- | --- |
| 1 | Deressa & Azazh, 2011 | Y | Y | Y | Y | Y | Y | Y | Y | Y | Y | 100 | Accepted |
| 2 | Teshome G, 2012 | Y | N | Y | N | Y | Y | Y | N | Y | N | 60 | Accepted |
| 3 | Teshome & Gedif. 2013 | Y | Y | Y | Y | Y | Y | N | Y | Y | Y | 90 | Accepted |
| 4 | Gebreslassie, et al. 2013 | Y | Y | Y | Y | Y | Y | N | Y | Y | Y | 90 | Accepted |
| 5 | Gebrehanna E et al. 2014 | Y | Y | Y | N | N | Y | N | Y | Y | Y | 70 | Accepted |
| 6 | Birhanu B, 2014 | Y | Y | N | Y | N | y | N | y | N | y | 60 | Accepted |
| 7 | Aklog T et al. 2013 | Y | Y | Y | N | Y | Y | N | Y | Y | Y | 80 | Accepted |
| 8 | Tsegay & Esmael. 2014 | Y | Y | Y | N | Y | Y | Y | Y | Y | Y | 90 | Accepted |
| 9 | Wondimu GA at al. 2017 | Y | Y | Y | N | Y | Y | N | Y | Y | Y | 80 | Accepted |
| 10 | Dachew BA et al. 2015 | N | N | Y | N | Y | Y | Y | Y | Y | Y | 70 | Accepted |
| 11 | Kebede Y, 2002 | Y | Y | Y | N | Y | Y | Y | Y | Y | Y | 90 | Accepted |
| 12 | Tesfaye G, et al. 2014 | Y | Y | Y | N | Y | Y | N | Y | Y | Y | 80 | Accepted |
| 13 | Deresse A, et al. 2014 | N | N | Y | N | Y | N | Y | N | Y | Y | 50 | Accepted |
| 14 | Reda A, et al. 2012 | Y | N | Y | N | Y | Y | Y | Y | Y | Y | 80 | Accepted |
| 15 | Reda A, et al. 2012 | Y | N | Y | N | Y | Y | Y | Y | Y | Y | 80 | Accepted |
| 16 | Reda A, et al. 2012 | Y | N | Y | N | Y | Y | Y | Y | Y | Y | 80, | Accepted |
| 17 | Dereje N, et al. 2014 | Y | N | Y | N | Y | Y | Y | Y | N | Y | 70, | Accepted |
| 18 | Kassa A et al. 2014 | N | Y | N | N | Y | Y | Y | Y | N | Y | 60, | Accepted |
| 19 | Kassa A et al. 2014 | N | Y | N | N | Y | Y | Y | Y | N | Y | 60 | Accepted |
| 20 | Astatkie A et al. 2015 | Y | Y | Y | Y | Y | Y | Y | Y | Y | Y | 100 | Accepted |
| 21 | Fufa G et al. 2017 | Y | Y | Y | N | N | Y | Y | N | N | Y | 60 | Accepted |
| 22 | Dires E, et al. 2016 | N | N | Y | N | Y | Y | Y | N | Y | Y | 60 | Accepted |
| 23 | Abdeta T et al. 2017 | Y | Y | Y | Y | Y | Y | Y | Y | Y | Y | 100 | Accepted |
| 24 | Abrha K, 2011 | Y | N | Y | N | Y | y | Y | Y | Y | Y | 80 | Accepted |
| 25 | Lakew A, et al. 2014 | Y | Y | Y | Y | N | N | Y | Y | N | Y | 70 | Accepted |
| 26 | Mekonen T et al. 2017 | Y | Y | Y | N | Y | Y | Y | N | Y | Y | 80, | Accepted |
| 27 | Adere A et al. 2017 | Y | N | Y | N | Y | Y | N | Y | N | Y | 60 | Accepted |
| 28 | Birhanu MA et al. 2014 | Y | Y | Y | Y | Y | Y | Y | Y | Y | Y | 100 | Accepted |

Note: Y: Yes, N: No

Each yes account 1 point (accepted if the score ≥50%)

Quality Assessment check lists

| S.no | Items | Response categories | | | |
| --- | --- | --- | --- | --- | --- |
|  |  | Yes | No | Unclear | NA |
| 1 | Was the sample representative of the target population? (Q1) |  |  |  |  |
| 2 | Were study participants recruited in an appropriate way? (Q2) |  |  |  |  |
| 3 | Was the sample size adequate? (Q3) |  |  |  |  |
| 4 | Were the study subjects and the setting described in detail? (Q4) |  |  |  |  |
| 5 | Was the data analysis conducted with sufficient coverage of the identified sample? (Q4) |  |  |  |  |
| 6 | Were objective, standard criteria used for the measurement of the condition? (Q6) |  |  |  |  |
| 7 | Was the condition measured reliably? (Q7) |  |  |  |  |
| 8 | Was there appropriate statistical analysis? (Q8) |  |  |  |  |
| 9 | Are all important confounding factors/subgroup/difference identified and accounted for? (Q9) |  |  |  |  |
| 10 | Were subpopulation identified using objective criteria? (Q9) |  |  |  |  |

NA: not applicable
