# Supplementary material for: Prevalence of lifetime substances use among students in Ethiopia: a systematic review and meta-analysis
Source: Syst Rev. 2019 Dec 14;8:326. doi: 10.1186/s13643-019-1217-z (PMC6911280; doi:10.1186/s13643-019-1217-z)
Supplement: Supplementary file 3 — Additional file 3: Table S3. Subgroup analysis of lifetime prevalence alcohol, khat and cigarette smoking among students in Ethiopia. [file 13643_2019_1217_MOESM3_ESM.docx]

Table S3: Subgroup analysis of lifetime prevalence alcohol, khat and cigarette smoking among students in Ethiopia

| **Subgroup** | **Khat** | | | **Alcohol** | | | **Cigarette smoking** | | |
| --- | --- | --- | --- | --- | --- | --- | --- | --- | --- |
|  | n | % (95% CI) | (Cochran Q, P-value) | n | % (95% CI) | (Cochran Q, p-value) | n | %(95% CI) | (Cochran Q, P-value) |
| By region  Somali  Tigrai  Oromia  Harari  Amhara  SNNP  Addis Ababa  Other | 1  2  5  1  10  2  1  - | 33.3(29.6, 37.3)  31.5(29.0, 34.0)  28.1(21.0, 35.8)  24.8(22.8, 26.9)  22.6(18.6, 26.8)  22.0(20.1, 23.9)  14.1(11.5, 17.1)  - | (55.6, 0.000) | 2  2  1  5  2  2  - | -  50.2(47.5, 52.9)  46.6(44.3, 49.0)  21.6(19.7, 23.6)  49.8(36.8, 62.8)  56.4(54.1, 58.6)  42.5(40.8, 44.2)  - | (25.1, 0.000 | -  2  1  1  6  1  1  1 | -  12.8(11.1, 14.6)  22.0(19.5, 24.7)  14.0(12.4, 15.7)  13.3(9.1, 18.1)  14.8(12.9, 16.9)  8.7(6.6, 11.2)  17.6(11.2, 24.1) | 222.6, 0.000 |
| Sample size  <500  500-1000  >1000 | 4  135 | 17.3(12.5, 22.7)  25.8(21.7, 30.1)  27.6(22.3, 33.2) | (8.1, 0.017) | 2  8  4 | 61.2(57.7, 64.7)  44.0(34.7, 53.6)  43.8(28.0, 60.3) | 14.2, 0.001 | 2  6  5 | 12.7(10.4, 15.1)  12.5(8.5, 17.2)  18.2(12.6, 24.5) | 3.2, 0.199 |
| Female proportion (%)  10-20  20-30  30-40  40-50  >50 | 3  5  7  7  - | 24.9(20.8, 29.3)  28.2(22.2, 34.6)  23.6(16.9, 31.0)  23.1(17.9, 28.8)  - | (1.6, 0.657) | 1  2  5  6  - | 48.6(44.5, 52.8)  55.6(53.5, 57.6)  47.6(32.6, 62.9)  42.2(30.5, 54.3)  - | 2.3, 0.309 | 1  2  4  5  1 | 13.1(11.2, 15.3)  17.9(16.4, 19.5)  12.9(7.8, 19.2)  12.7(8.8, 17.3)  28.6(26.5, 30.9) | 56.5, 0.000 |
| Education level  Secondary school  University/college | 4  18 | 22.5(15.2, 30.7)  25.1(21.9, 28.5) | (0.4, 0.544) | 3  11 | 41.4(22.1, 62.1)  47.8(39.9, 55.7) | 0.3, 0.570 | 3  10 | 21.5(12.6, 32.1)  12.9(10.1, 16.0) | 3.1, 0.078 |
| Mean age (year)  <20  20+ | 5  17 | 20.5(13.8, 28.2)  25.9(22.6, 29.3) | (1.6, 0.201) | 4  10 | 46.0(28.6, 63.9)  46.6(38.3, 55.0) | 0.0, 0.950 | 4  9 | 17.7(9.8, 27.3)  14.7(11.3, 18.5) | 0.8, 0.364 |
| Year of publication  2000-2010  2011-2014  2015-2017 | 1  13  8 | 26.7(24.1, 29.4)  26.2(22.3, 30.4)  21.9(17.6, 26.5) | (3.3, 0.195) | -  10  4 | -  44.9(35.7, 54.3)  50.1(37.9, 62.4) | 0.4, 0.510 | 1  10  2 | 13.1(11.2, 15.3)  15.7(11.4, 20.5)  12.3(10.8, 13.8) | 2.3, 0.319 |
| Study year  2000-2010  2011-2014  2015-2017 | 3  14  5 | 21.7(15.4, 28.7)  26.3(22.6, 30.3)  21.9(15.5, 29.0) | (4.9, 0.084) | 3  10  1 | 32.4(17.5, 49.3)  52.1(44.6, 59.6)  33.1(29.5, 36.9) | 20.1, 0.000 | 3  9  1 | 12.0(9.2, 15.0)  16.6(12.1, 21.7)  7.9(6.0, 10.3) | 13.6, 0.001 |

Cochrane Q: Cochrane Q test for heterogeneity statistics for subgroup, P-value: P-values for significancy test
